# Review of Shoreline Extraction Methods from Aerial Laser Scanning

**DOI:** 10.3390/s23115331

**Published:** 2023-06-04

**Authors:** Andrzej Stateczny, Armin Halicki, Mariusz Specht, Cezary Specht, Oktawia Lewicka

**Affiliations:** 1 Department of Geodesy, Gdańsk University of Technology, Gabriela Narutowicza 11-12, 80-233 Gdańsk, Poland; 2 Marine Technology Ltd., Wiktora Roszczynialskiego 4-6, 81-521 Gdynia, Poland; a.halicki@marinetechnology.pl (A.H.); or m.specht@wn.umg.edu.pl (M.S.); 3 Department of Transport and Logistics, Gdynia Maritime University, Morska 81-87, 81-225 Gdynia, Poland; 4 Department of Geodesy and Oceanography, Gdynia Maritime University, Morska 81-87, 81-225 Gdynia, Poland; c.specht@wn.umg.edu.pl (C.S.); o.lewicka@wn.umg.edu.pl (O.L.)

**Keywords:** aerial laser scanning (ALS), light detection and ranging (LiDAR), shoreline detection, shoreline extraction

## Abstract

Autonomous technologies are increasingly used in various areas of science. The use of unmanned vehicles for hydrographic surveys in shallow coastal areas requires accurate estimation of shoreline position. This is a nontrivial task, which can be performed using a wide range of sensors and methods. The aim of the publication is to review shoreline extraction methods based solely on data from aerial laser scanning (ALS). This narrative review discusses and critically analyses seven publications drawn up in the last ten years. The discussed papers employed nine different shoreline extraction methods based on aerial light detection and ranging (LiDAR) data. It should be noted that unambiguous evaluation of shoreline extraction methods is difficult or impossible. This is because not all of the methods reported achieved accuracy, the methods were assessed on different datasets, the measurements were conducted using different devices, the water areas differed in geometrical and optical properties, the shorelines had different geometries, and the extent of anthropogenic transformation. The methods proposed by the authors were compared with a wide range of reference methods.

## 1. Introduction

A shoreline, which is the dynamic boundary line between land and water [1,2], is subject to changes in time and space [3,4,5]. Information about the shoreline position and its changes play a crucial role in numerous applications such as coastal zone management [3,6], autonomous navigation [7,8,9], hydrodynamic modelling [10], engineering [11], urban planning [11], ecosystem monitoring [11,12], and disaster management [11,12,13], as well as climate change studies [11,12]. This highlights the significant impact of accurate shoreline data in diverse fields such as economics, engineering, and science [1,3].

As the shoreline changes over time, it is necessary to conduct regular shoreline measurement [14,15,16]. The surveying methods can be diverse, including aerial and historical photos, maps, in situ measurements, and surveys carried out from remote sensing platforms using optical devices [1,11,17,18,19]. The most widely employed methods include traditional topographical surveys, such as global positioning system (GPS) real-time kinematic (RTK) methods. Such methods allow for precise point-to-point data but are limited by their speed. In other words, when measuring instantaneous shoreline, such measurements provide results from different timesteps. As such, it may not be best suited for some applications, or for comparison with satellite-derived data. On top of that, those methods require extensive human effort and are difficult to carry out in areas with limited access (e.g., marshes).

Optical systems are often used in shoreline extraction studies. Terrestrial video measurements offer good area coverage, but require significant processing (orthorectification, georeferencing with use of ground control points (GCP)). Aerial photogrammetric surveys have similar characteristics, providing coverage even for large study areas. Additionally, those are typically more costly, especially when aeroplanes are involved. They are also weather-dependent, can only be performed during daylight, and, in contrast to light detection and ranging (LiDAR) techniques, provide 2D information, unless heavily pre-processed (e.g., by using structure from motion techniques, which also requires careful flight planning).

Satellite remote sensing, especially with the latest generation satellites, has increasingly become the preferred choice for shoreline extraction due to its high resolution and lowering the costs of data acquisition. High- and very-high-resolution satellites expedite data acquisition and processing, offering detailed information and comparable precision to traditional photogrammetric aerial acquisitions. However, they have similar limitations as those discussed above. An alternative sensor that can be used for satellite shoreline detection is synthetic aperture radar (SAR) [2], which is not weather- or daylight-dependent. One of the greatest drawbacks of the satellite techniques is the requirement of the satellite covering the area. Thus, the researchers are limited by satellite revisit times.

**Figure 1:**
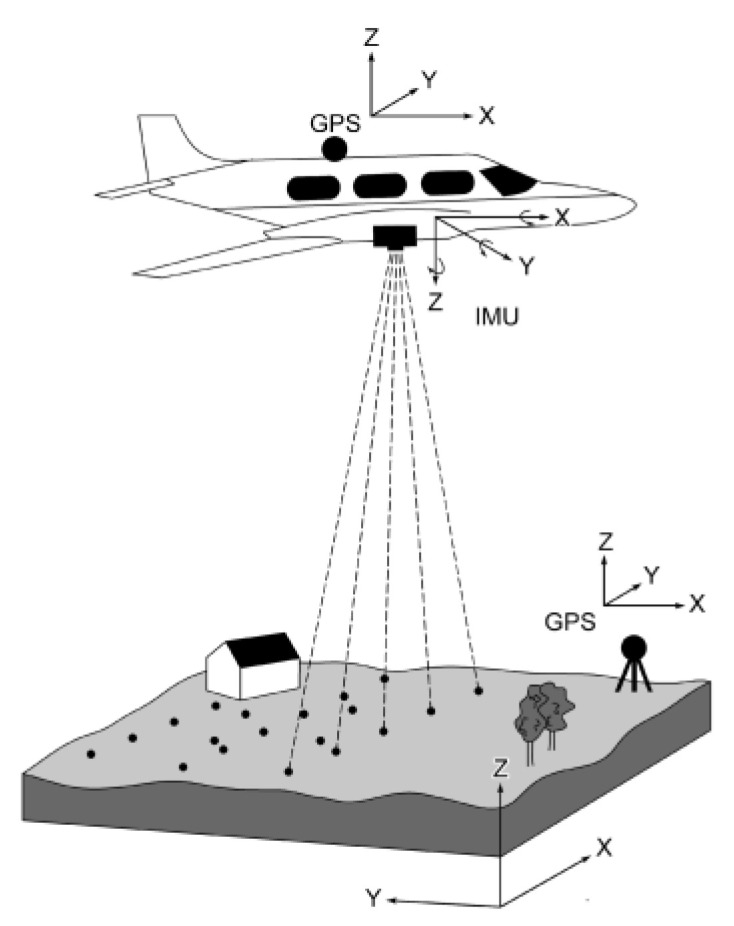
Illustration of aerial LiDAR measurement with a highlight on the need of georeferencing data by GPS and inertial measurement unit (IMU) reference frames, as well as the global reference frame.

LiDAR techniques are particularly useful tool for shoreline measurements, as they allow for large areas to be covered in a relatively short time [1,20]. This includes both terrestrial laser scanning (TLS) and aerial laser scanning (ALS), both of which are interesting methods for shoreline data acquisition. They are increasingly affordable [21] and daylight-independent [20]. When mounted on mobile platforms, those methods require georeferencing (Figure 1), but generally can provide a three-dimensional overview of the area without extensive pre-processing (i.e., structure from motions techniques). Furthermore, LiDAR can be mounted on board a relatively small unmanned aerial vehicle (UAV), allowing for easy transportation and convenient deployment of the sensor, whenever the researcher finds it necessary. As such, multiple flights can be performed in a short time scale, allowing for short-term studies, which is difficult to achieve with satellite-derived data. Currently, the biggest drawback of LiDAR surveys is the amount of gathered data. Given the increasing demand for accurate shoreline data and the amount of collected data, there is a growing need for automated extraction methods.

This paper provides a comprehensive review of shoreline extraction methods based on ALS. Consequently, the data examined in these studies were collected during LiDAR measurements carried out from aerial vehicles, such as aeroplanes and drones. In most cases, the data collection was not conducted in the manuscripts; instead, available repositories were used. All of the articles included in the review were published during the last ten years (i.e., in the years 2011–2021).

This review is structured as follows: Section 2 describes data sources, the current state of knowledge, and criteria for publication selection in the field of shoreline extraction. Section 3 presents nine shoreline extraction methods, such as anthropogenic, contour, cost optimisation, elevation gradient trend propagation (EGTP), grid, morphological, object-oriented, and profile. The publication concludes with a comparative analysis of the aforementioned methods and a discussion on general and detailed conclusions.

## 2. Materials and Methods

### 2.1. Data Sources

This publication is a subjective review of shoreline extraction methods. However, the authors of this review attempted to conduct the search in a way that would be as systematic as possible, with the aim of facilitating its repetition. The Google Scholar search engine and the ResearchGate portal were used to search for scientific publications. The search was performed using keywords: ALS, LiDAR, shoreline detection, and shoreline extraction, as well as their combinations.

### 2.2. Current State of Knowledge and Paper Contribution

During writing of this manuscript, to the best of the author’s knowledge, no reviews of the literature were dedicated exclusively to ALS-based shoreline extraction, which was the main motivation for this publication. However, shortly before manuscript submission, a comprehensive review of the literature by Wang et al. [22] was released on this topic. While both studies are concerned with the same area and contain some overlapping works, this manuscript delves deeper into certain aspects—the details of individual methods, reference methods used, the importance of standardisation, and open science in this area of research, as well as the significance of minimising the need for human supervision of the extraction process.

This work provides fresh insights by using concrete examples from a carefully chosen subset of recent papers to support generalised observations, highlighting advancements in the last decade. Contrasting with the broad scope of [22], the refined focus of this paper allows a meticulous critique. We stress the need for shared methods and codes, augmenting [22] emphasis on public datasets. Moreover, we underline the importance of standardised methods and datasets, enhancing reproducibility and comparability in this field. Collectively, these contributions offer a unique perspective for future shoreline extraction research.

### 2.3. Criteria for Publication Selection

The methods discussed in this review were accepted or rejected based on the following criteria:

- The method must perform shoreline extraction from either a digital elevation model (DEM)/digital terrain model (DTM) or a point cloud. This means that the methods that do not directly lead to obtaining a shoreline are to be rejected. Examples of such methods can be found in a study by Smeeckaert et al. [23], which presents a method for classifying water areas based on LiDAR, or in a study by Xu et al. [24], which proposes a method for bridge extraction;
- The proposed method must use exclusively ALS data;
- The above means that the methods based on a sensory fusion are to be rejected, even if the sensory fusion includes ALS;
- The method can use other sources of information in order to extract the shoreline, as long as the information is not derived directly from other sensors (e.g., camera). Such information could include the tidal reference system or the orientation of the shore;
- If a particular paper proposes several methods, further analysis takes into account only the methods using the ALS data exclusively;
- Due to the rapid development of geoinformatics and computational sciences, the proposed method had to be published within the last ten years. Thus, the literature review covered the years 2011–2021.

## 3. Results

### 3.1. General Overview

This review discusses and critically analyses seven papers drawn up in the last ten years, which applied nine different shoreline extraction methods, most of which were original authors’ methods or modifications of the existing ones. Among the analysed papers, there are those that carried out very extensive validation of the method using many different types of shoreline and waterbodies with different geometric and optical characteristics (e.g., [25]), as well as those in which the method is only tested on a single dataset (e.g., [26]). A cumulative list of all the papers discussed as part of the conducted review is provided in Table 1.

Most of the discussed papers relate the errors obtained in the shoreline extraction process using their own method to the results obtained using the methods proposed by other researchers or other reference methods. Only a study by Yousef et al. [30] does not compare the proposed extraction method with any other reference method, as it was limited to a visual analysis of the result. However, in a later study conducted by the same authors [31], quantitative analysis of results was performed. The proposed methods were compared with two reference methods and the Monte Carlo simulation was used in order to estimate the mean error of methods.

It is worth noting that many papers compare the accuracy achieved on completely different datasets, which prevents an unambiguous assessment of the methods being compared. This indicates the need for meta-analyses in this area, as well as the importance of reference methods standardisation. Additionally, it shows the need and significance of creating methods in publicly available software, which greatly facilitates independent implementation of shoreline extraction methods without an advanced programming background [27].

The main assumptions underlying each of the individual methods discussed in the study can be found in Table 2. Additionally, the table below also contains the main operations performed during the shoreline extraction process. It can be noted that most of the examined extraction methods use information acquired based on the local tidal system, as it allows for effective shoreline contouring. Only the cost optimisation model method proposed by Xu et al. [25] does not use the tide level data. Although the aforementioned method uses the threshold value for the height of a particular point, this is calculated in relation to the nearest fitted plane and not to the water level. The other methods use the assumptions based on the less frequent occurrence of reflections from water areas and the water surface flatness.

Information on sharing methods and sources of data used in each of the discussed studies is provided in Table 3. Sharing methods in any form (i.e., as plugins, source codes, or stand-alone executables) is critical to the establishment of comparative studies. Furthermore, it improves the usability of the method to third parties (e.g., industry and other researchers). Openly sharing source code used in a study ensures correctness of method implementation. Moreover, code sharing can facilitate the creation of community driven improvements to the proposed method. Most of the studies shared the sources of their data. Source code was not shared in any of the papers. Only Liu et al. [29] shared all of the methods used in their study, which was an ArcGIS software extension called “ShorelineExtractor”. Additionally, the contour method, the first of the three methods investigated by Farris et al. [27], was described by the authors in an extensive way, which allows any ArcGIS user to easily execute it manually. In the same paper [29], the importance of method and code sharing for third party usability was underlined, and the authors declared that their future aims include sharing all of their methods in a user-friendly way.

### 3.2. Contour, Grid and Profile Methods (Farris et al., 2018 [27])

The study compared the methods used by the USGS under the Marine Geology Program. The three methods used were described in great detail and are referred to as the contour, grid, and profile methods. When comparing the above methods, observations were made that prompted the authors to divide the contour and profile methods into internal variants. This means that the study compared a total of seven methods. However, all of them were based on three basic methods (Table 4). The shoreline extraction was performed based on two datasets derived from ALS of areas with extremely varying shoreline geometry. The data were derived from measurements conducted by the NOAA and USGS. They were downloaded from NOAA repositories. The authors provided the exact source of the data, but the URL links leading to the repositories had expired. The data were recorded using the ATM-2 system.

**Figure 2:**
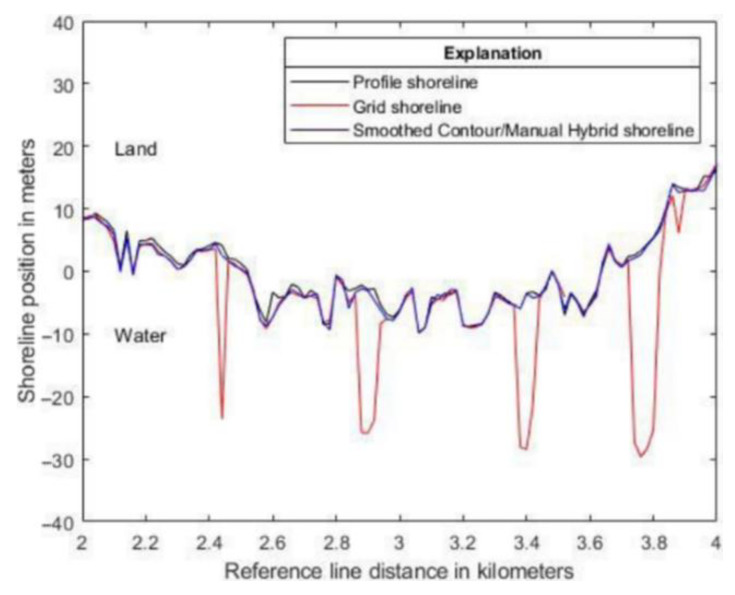
Comparison of the grid method, profile method, and the smoothed contour/manual hybrid method. The shoreline resulting from the use of the last method was obtained by smoothing the contour method, followed by manual editing and smoothing using a line smoothing tool in the ArcGIS software. The *x*-axis represents the distance along the shore. The *y*-axis represents the shoreline position. The position of the shoreline is provided in relation to the reference line. The obtained shorelines were rotated to facilitate the comparison of the differences [27].

Unavailability of the actual position of the shoreline prevented the comparison of the extraction errors. For this reason, the paper compares the differences in the results of the extraction carried out by the individual methods for both datasets. It is quantitatively demonstrated that for a straight shoreline, the results of shoreline extraction are very similar. It can be seen by comparing the mean differences in shoreline positions, RMS differences in shoreline positions (Table 5), and RMS differences in shoreline positions after the mean difference is removed. Visual comparison of shoreline extraction on a complex shoreline was also provided (Figure 2)

Based on the obtained results, the paper provides strengths and weaknesses of compared methods (Table 6). One of the key differences between the methods concerns the way the uncertainty of the shoreline position is assessed. Both the grid and profile methods ensure a point-based assessment of uncertainty (analogous to [25]), while the contour method only enables a summary estimation of uncertainty. Another difference, which was only qualitatively assessed in the publication, concerns the effect of data quality on the results obtained. In situations such as the occurrence of several heights for a single point (cell) due to having made several flights over the water area, the occurrence of a water level exceeding the MHW level, and complex shoreline geometry, the results of the application of the methods concerned require a supervised quality control process. This is particularly time-consuming for the profile method, for which it is necessary to carry out a visual inspection of each profile. The grid method was described as the fastest one, yet it was pointed out that where this method was applied, it was necessary to develop more complex quality control methods.

Moreover, the extent to which a particular method could be disseminated was assessed in the paper. Two of the three proposed methods require advanced knowledge of a programming language due to the need to implement them independently. Only the contour method uses the ready-made tools available in the ArcGIS software. Hence, the contour method was rated as the easiest to transfer to other users. Unfortunately, the paper does not share the source code used to develop the grid and profile methods. However, it mentions that it is planned to streamline the above methods and to share them in the future in a form that is openly accessible and user-friendly.

### 3.3. Anthropogenic Method (Hua et al., 2021 [28])

**Figure 3:**
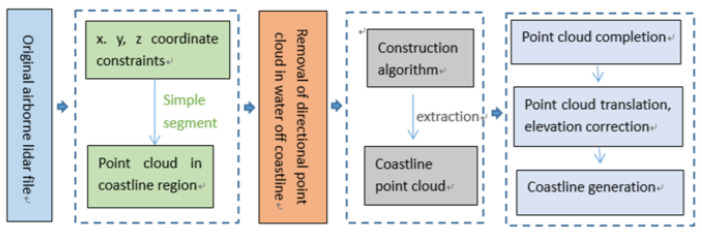
A diagram of shoreline extraction proposed by Hua et al. [28].

This article addressed the issue of detecting shores of an anthropogenic nature. At the very beginning of the paper, the problem related to the volume of data derived from LiDAR measurements is addressed. Simple criteria are proposed that enable the rejection of a portion of the point cloud, thus, reducing the computational complexity at the later stages (Figure 3).

External software was used for the point cloud visualisation and analysis to calculate the ranges of coordinates for the entire area, the range of coordinates in which the shoreline is located, and the direction of taking the scan, as well as to determine on which side of the scan the shoreline was located. Segmentation of the area was also performed using the third party tools. Unfortunately, the paper does not specify exactly what program was used during the above stages. In the next stage, points derived from the reflections on the water surface were removed. The paper does not describe the method used. The reader is informed that in order to detect the points reflected from the water surface, either the low values of the reflection intensity, the low heights of the points, or the degree of isolation of the point cluster could be used. Subsequently, the shoreline extraction process begins. To this end, information about the scan’s flight direction is used. On that occasion, it is emphasised that the information on the scan’s flight direction could be acquired based on the analysis of changes in the geographic coordinate values during the recording. This observation, while pertinent, appears to be quite obvious.

As for the set of data used in the study, the flight proceeded from east to west. Based on the above, and using the knowledge that the shoreline subject to extraction is the northern edge of the area, the task was to find the northern-most point in the cloud. To this end, a mathematical formulation was developed, which utilised the basic properties of geographic coordinates. The northern-most point can be characterised by a lower latitude than the points located before it or after it (when indexing the points in the cloud during the recording). In order to “increase the algorithm’s stability”, arbitrary criteria were adopted. The first criterion involves checking whether a point located six indices away also has a higher latitude. This procedure intends to avoid a local minimum. This (very arbitrary) criterion was described using the set of equations below (Equation (1)):

(1) $$\begin{array}{c} p_{i}\cdot y<p_{i-1}\cdot y\\ p_{i}\cdot y<p_{i+1}\cdot y\\ p_{i}\cdot y<p_{i+6}\cdot y \end{array},$$

where *p* denotes the point cloud outside the shoreline and *y* is the coordinate of a point.

The second criterion is the difference in the latitudes of the point and the point preceding it. The value of the above-mentioned difference must exceed the adopted threshold value. Again, this procedure intends to avoid the local minimum by ensuring that the points being compared are not located too close to each other.

**Figure 4:**
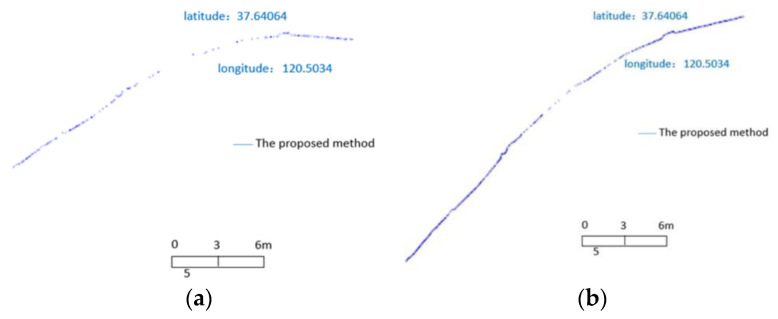
Original shoreline point cloud determined using the anthropogenic method (**a**) and point cloud after completion (**b**) [28].

The first of the point cloud operations in the algorithm is the point cloud densification. The paper proposes its own densification method based on an adaptive method. The results achieved with the novel method are shown below (Figure 4). Unfortunately, the description of the method used is not complete, and the method itself is far from intuitive. The method for densifying the obtained point cloud involves the addition of three, five, or eight points between the analysed pair of points belonging to the shoreline. The decision on the number of added points is taken based on the $d_{p_{i}p+1}$ variable value. However, the paper does not clearly indicate the meaning of the variable in question. It also seems to lack a concise mathematical form to enable an independent analysis. The variable $d_{p_{i}p+1}$ can be the distance separating two neighbouring points of the isolated shoreline (more precisely: a cloud of shore points). Nevertheless, with such an interpretation of the above-mentioned variable, further considerations in the manuscript become unclear when (depending on whether the value of $d_{p_{i}p+1}$ is higher than 1) it falls within the range from 1 to 2 (with the paper failing to indicate, on that occasion, that the second condition is, so to speak, a subset of the first condition), or is higher than 2 (same applies). Presumably, the first condition was intended to check whether $d_{p_{i}p+1}$ is lower or equal to 1 and not higher than 1; at least, this is how the authors of this review interpret it. When distributing the points added to the point cloud, an adaptive algorithm is used. Its behaviour is calculated by the number of added points (i.e., determined by the value $d_{p_{i}p+1}$). If three points are added, these are distributed at coordinates located at 1/4, 2/5, and 3/4 of the distance separating the original pair of points, respectively. Similarly, if five points are added, these are distributed at 1/8, 1/7, 1/5, 2/5, 1/2, and 3/4 of the distance separating the original pair of points. If eight points are added, the proportions used when calculating their positions are 1/8, 1/7, 1/5, 2/5, 1/2, 3/5, 3/4, and 4/5. As stated in the manuscript, the values were selected arbitrarily, and their selection was intended to smooth the obtained shoreline.

**Figure 5:**
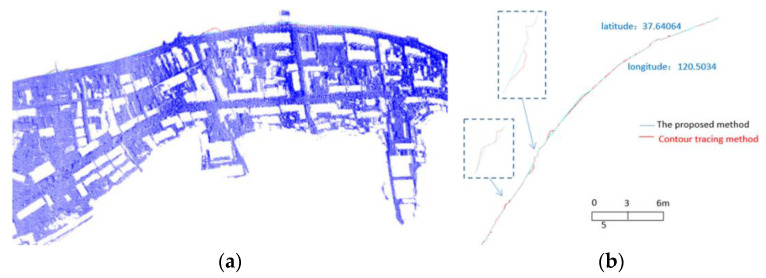
Superposition of anthropogenic and contour tracking methods and original cloud (**a**), as well as the effect of superposition comparison of two methods (**b**) [28].

The steps described above were used to obtain a densified shoreline in the form of a point cloud. The densification result is presented above. The proposed method was compared with the contour method. Unfortunately, there seems to be no source of the reference method specified in the paper. There also seems to be no description of the method applied. Therefore, it is not certain what method is used exactly, although it is likely that either the method described by Farris et al. [27], or the one proposed by Liu et al. [29] is involved. A validation of the extraction results is limited only to the visual comparison of the results (Figure 5).

### 3.4. Contour and Object-Oriented Methods (Liu et al., 2011 [29])

In the article [29], an extensive analysis of the algorithmic routines that can be used for shoreline extraction algorithms based on LiDAR data and imaging is presented. Software that enables easy implementation of all the methods discussed in the paper was developed and shared. These are available in the form of an ArcGIS plugin called “ShorelineExtractor” [29].

**Figure 6:**
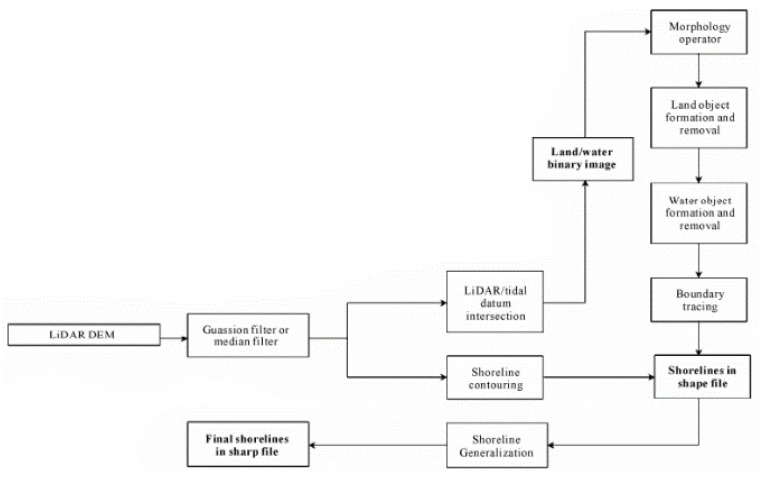
Diagram of algorithm stages in the object-oriented method (the upper branch on the first fork) and the contour method (the lower branch on the first fork) [29].

As only LiDAR data fall within the thematic scope of this review, the focus is solely on the methods based on LiDAR. A diagram of the algorithm for LiDAR data is presented in Figure 6. The diagram includes two methods described in the study, i.e., the object-oriented method and the contour method [20,27]. In the object-oriented method, a cluster of land or water pixels is regarded as an object, while the shorelines are established as boundaries between the clusters of different classes. For the validation of the methods operating on the LiDAR data, the authors used the data collected using an ATM sensor. The authors performed a projection for the universal transverse Mercator (UTM) 15N reference system, horizontally referenced to the World Geodetic System 1984 (WGS 84) ellipsoid, with the North American vertical datum of 1988 (NAVD 88) vertical reference system.

For both methods, filtration of the obtained DEM using a moving window with dimensions of 3 × 3 pixels is performed. Then, using the object-oriented method, the common parts of the DTM and the tidal system are found, thus, dividing DEM cells into land and water cells based on the MHW level. The segmentation result is subjected to morphological operations based on the opening and closing operations. Consequently, the boundaries are smoothed, and small parts of objects resembling bays or peninsulas, which are, in fact, segmentation errors, are removed. Afterwards, objects are detected. To this end, observations about the continuity of land and water masses are used. The linked pixels of a particular category are combined into larger objects using the recursive expansion algorithm. Two pixels are defined as connected if they are in close proximity to each other. The entire raster is scanned row-by-row, with the first land pixel found becoming the seed and is regarded as a land object. The expansion of a single pixel is then performed until all the connected pixels of the same category are found inside the object. This process can be recursively repeated in order to find more objects. Moreover, filtration is performed based on the object’s surface in order to remove small objects that may interfere with the course of the obtained shoreline. The removal involves changing the class of the object to the opposite one. The shoreline pixels are then identified by scanning each land pixel using a moving window with dimensions of 3 × 3. If one or more of the neighbouring pixels belongs to the water pixels, the examined pixel (found inside the moving window) is marked as a pixel co-forming the shoreline.

A second extraction method is also employed, which is referred to as the contour method. The height of the tidal reference system is detracted from the height in the DEM. Contours are then created using the cells with a height equal to zero. Shoreline obtained by this method requires manual cleaning, which is time-consuming. The paper aims to solve that problem by proposing an automatic, parametric solution. The modified contour method performs the filtration of the shoreline components based on the section’s length threshold value. The application of parametric filtration methods in both methods allows noise to be eliminated by reducing the extraction error. The presented results were obtained for different parameter settings, thus, demonstrating the possibilities of noise elimination. As the methods were tested on a single dataset, the robustness of parameters to the specificity resulting from the individual characteristics of a particular dataset is unknown. Therefore, it is not known whether it is necessary to parametrise the method each time or whether it is possible to establish universal parameters.

For both extraction methods, two options of generalisation and smoothing of the obtained shoreline are available. Both can be performed in the developed software tool by selecting the appropriate option. The first generalisation method is shoreline simplification using the Douglas–Peucker method [33]. This approach retains critical points, which are particularly important for maintaining the basic shape of the curve, by removing redundant points that produce smaller deformations. The algorithm connects the end lines of the curve using a trend line. The algorithm is effective and reduces the volume of the obtained data, while the resultant shoreline may include acute angles, which limits its quality in cartographic and hydrographic applications. The second method involves an analysis of the shoreline shape and the elimination of bends with high curvature. Some of these are removed, and others are subject to correction by expanding in order to satisfy the adopted tolerance threshold. The authors note that when compared to the Douglas–Pecker method, this method produces smoother shorelines of better quality in terms of cartographic and hydrographic applications. The authors also implemented the methods in such a manner that the user can select one of them when initialising the shoreline extraction process. The only parameter that must be provided by the user is the screening parameter that determines the degree of generalisation.

### 3.5. EGTP Method (Luque et al., 2012 [26])

In this article, a shoreline extraction method called the EGTP is presented. The approach is based on the use of an iterative method on a rectangular grid. The height gradient trend is calculated for each grid cell of a known height towards the cells of an unknown height. The process is repeated until the new point of the grid reaches a level similar to (but lower than) the selected vertical reference system.

In order to determine the reference line (zero height), transverse profiles were calculated using the DSAS [34]. On this basis, a reference shoreline was determined using the method proposed by Stockdonf et al. [20], which was also discussed in another study by Farris et al. [27]. The discussed paper used a buffer on both sides of the profile in order to record the distance to the data from the profile and the height for each transverse profile. Then, the linear regression by the least square method was used to calculate the coefficients in the equation of a straight line. Subsequently, the intersection between the fitted regression line and the selected water level or reference system was determined. The uncertainty of the reference line position was calculated for each profile using the covariance matrix.

In the first step of the proposed algorithm, points with elevation lower than the threshold value (in relation to the reference height) are removed. For the remaining points, the initial local elevation gradients and their uncertainties in the N–S and E–W directions are determined using the Sobel filter. The gradients are calculated using the convolution operation for the neighbourhood with dimensions 3 × 3. Then, based on the error propagation rule, the gradient uncertainties are determined. The determination of the gradient for a particular step only takes place for the points that have a full neighbourhood (i.e., are surrounded by eight other DEM cells). The height gradient for the points located on the boundaries of these grid cells is interpolated by the inverse distance weighting (IDW) method using the gradients calculated for the nearest neighbouring grid points. For the gradients determined in this way, uncertainty is calculated as well. The elevation values are then extrapolated for the cells with an unknown value. A weighted average determined using a movable window with dimensions 3 × 3 is used. The cell height is calculated based on the determined gradients and the elevations of the neighbouring cells, while taking into account the position calculated in relation to the central cell. The process is locally stopped at the moment when the estimated heights are below the required level in relation to the zero elevation. The authors apply the local process stopping when the extrapolated height values are negative values. Similar to the gradients, uncertainties for the extrapolated elevations are estimated while taking into account the uncertainty of both the terrain model and the extrapolation process. The last stage of the algorithm is the extraction of the shoreline contour from the extrapolated terrain model using the magnitude and direction of the local height gradient.

The proposed method was compared against the widely used transverse profile method proposed by Stockdonf et al. [20,27], which involves the linear fitting of the altimetric profiles. As this is one of the most widespread methods of shoreline extraction based on DEM, this approach can facilitate comparison with other results found in the literature. As compared to the profile method, the EGTP method proposed by the authors is presented as more robust and less supervised. The EGTP method does not require an elevation range, only the minimum elevation based on which the original DEM was created.

The authors presented an extensive method validation in which they compared the results of applying the EGTP method, the profile method, and the combination of both. They also compared the uncertainties obtained for the entire area using each method. For the EGTP method, the mean uncertainty was 2.08 m, while the median uncertainty was 1.51 m, which proved to be values higher than those for the reference contour method. However, as noted in the article, when performing the EGTP method, no outliers removal was performed, which may have negatively affected the results.

### 3.6. Cost Optimisation Method (Xu et al., 2019 [25])

This article proposes a parametric method for shoreline extraction based on point clouds. The first stage of the method in question is to reject the points belonging to water areas. To this end, detection of reflections from the water surface is used, as proposed by [24]. In the above-mentioned study, a method for detecting bridges in the LiDAR point cloud was developed. To this end, a plane fitting method by the RANSAC was used. The basic assumption in identifying the water surface is that the recorded water reflections should create a plane. High wave conditions can be a potential obstacle when using the method in question.

The proposed method tries to refine the first step by verifying whether the obtained planes actually belonged to water areas. To this end, the height and density characteristics proposed in a paper by Smeeckaert et al. [23] were calculated. In the original study, these characteristics were used for the training of the support vector machine (SVM) binary classifier that allows the points from the LiDAR cloud to be assigned to either the land class or the water class. The use of information on the density of obtained LiDAR reflections is a frequently used procedure when attempting to classify areas into water and land areas [23]. It is due to the fact that the signal reaching the water surface is, to a large extent, absorbed. However, this is not true for shallow and muddy water areas, where the occurrence of more reflections from the water surface is possible. Subsequently, the classification results are corrected. False points classified as water points are removed based on the information on the height above the plane and the density. If the height of a point belonging to the water class, measured above the fitted plane, is greater than the threshold *T_e_*, the class of the point is changed, and the point is classified as a land point. Moreover, if the density of points of the isolated plane exceeds the threshold value *T_d_*, the entire region is reclassified to include it in the land class. The values of the above-mentioned algorithm parameters are set by the user. Values used in the study are provided, with a remark that the proposed method may require parametrisation for a specific waterbody. It is also pointed out that the minimum dimensions of the waterbody for successful extraction should not be smaller than 500 m × 500 m. After performing the correction of the point classification, the waterbodies are removed. Then, the remaining points are clustered. To this end, the authors use the Euclidean clustering method [35,36] to divide disjoint areas into individual clusters.

The next step is to isolate and optimise the resulting edge (shoreline). Initially, all points are regarded as indeterminate. For each indeterminate point, its *k*-closest neighbours are selected. Then, a convex hull of the obtained set of points is constructed, and all of the points found within the hull are labelled as non-boundary points. The process is repeated until a non-boundary point cannot be found. In such a case, all the points that retain the label of an indeterminate point are regarded as boundary points. In addition, the criterion of selection of the obtained boundary points based on the threshold value *T_d_* is applied. If a point marked as a boundary point is located at a distance longer than *T_d_* from the remaining boundary points, it is rejected. Several important issues related to this stage of the algorithm are mentioned in the manuscript. Choosing too high a value for the *k* parameter may result in certain boundary points being omitted. A low value of the *k* parameter ensures a more complete identification of boundary points while, however, increasing the computational complexity and, thus, the process duration. Moreover, it is possible to generate many potential boundaries from the set of points obtained with such an approach.

The paper aims to solve the problem by developing a novel cost function that should be subject to minimisation. The formula for the boundary cost *β* is defined as follows (Equation (2)):

(2) $$\beta =\sum_{i=1}^{n}\left(D\left(B_{i}\right)+\sum_{\left\{B_{i},B_{j}\right\}\in N}\lambda \left|\cos\left(\frac{\langle B_{i},B_{j}\rangle }{2}\right)\right|\right),$$

where *β* denotes the cost of obtaining the boundary, *n* indicates the number of links found in the boundary, *D(B_i_)* is the length of a particular link *B_i_*, *λ_i_* is a coefficient serving as a weight, *N* is the set of adjacent links, and *<B_i_,B_j_>* is the angle formed between the links *B_i_* and *B_j_*.

The boundary establishment cost defined this way is proportional to the length of particular sections and the angles formed between the neighbouring sections that make up the boundary. The *λ* coefficient is a weight for the term related to angles and serves the role of another parameter in the method under discussion.

The number of possible permutations for a real-world dataset is huge (for similar problem instances see, e.g., TSP problem described by Beasley in [37]). The authors use the backtracking approach to break down the optimisation in order to construct a global solution based on partial solutions that are iteratively evaluated. If the optimal path from the source to each node from the first column is known, then the path to the node from the second column should include the optimum path to the previous node. Then, the minimum cost path is determined from the end, i.e., from the sink to the source. The shoreline obtained in this manner is still subject to minor improvements. Threshold distance *T_b_* is used in order to remove the points that are located too far from the identified waterbodies.

To assess the accuracy of their method, completeness and correctness indicators were used. The average completeness was 92.5%, while the correctness was 90.7%, as calculated for all five datasets. The accuracy levels achieved using the proposed method were compared against four other studies (Table 7). The other studies used different datasets. The accuracy of the reference studies ranged from 1.5 m to 31 m, as compared to the level of 1 m achieved by the proposed method. On this occasion, it was stressed that a comparison of the point accuracy indicators was impossible, as the authors of the reference studies failed to assess the position accuracy of the obtained points. In the first two studies, the extraction was performed based on aerial photos. The other two studies were based on point clouds obtained using a LiDAR sensor. All four reference methods achieved a lower accuracy level than the proposed method. It is worth noting that the discussed method was tested on the largest number of datasets. It should also be noted that the results need to be regarded with some caution, since the comparison concerns the accuracy levels achieved on different datasets.

The issue of parametricity of the method was also addressed in the paper. The algorithm’s operation was tested for different parameter values (Table 8). All results presented in the article were obtained using the values presented in the third column of Table 8. It is worth noting the results were obtained using the same parameter settings on all datasets.

### 3.7. Morphological Method (Yousef et al., 2013a [30])

Two shoreline extraction methods are proposed in the article [30]. The first, referred to as the morphological algorithm, uses only the DEM developed based exclusively on ALS data. The second method, which uses the SVM, is based on a sensory fusion of LiDAR data and aerial photos. For this reason, only the first of the methods was included in this review. The morphological algorithm approach can be divided into eight main stages.

The authors used the previously prepared DEMs downloaded from NOAA repositories. These were prepared from the data gathered using the Riegl LMS-Q680i airborne LiDAR sensor, with the recording being performed in the UTM 18N system. Despite having used the previously prepared elevation models, the authors also described the overall process of transforming a point cloud from a LiDAR to DEM, which may help other researchers to carry out the process of result replication. It is ambiguous whether the terrain model used was prepared in exactly the same way (i.e., interpolated by the IDW method), but such an assumption appears to be justified.

To estimate missing values in the DEM, a locally weighted nonparametric regression [42] was performed. A window of a size four times larger than the missing value region, centred on that region, was used. Then, the local tidal system was established using the data on the MHW level during the epoch. These data were derived from the NOAA current and tide database. Both the DEM and the tidal system were brought to the NAVD 88 vertical reference system.

The next step of the morphological algorithm is the segmentation (i.e., classification) of the DEM cells into one of two classes. To this end, thresholding was used, with the local tidal reference system level adopted as the threshold value. All of the points located above the MHW level were classified as land points, and the remaining as water points.

Subsequently, the algorithm performed detection and removal of anomalies interpreted as measurement errors. Water area heights were assumed to be normally distributed. Afterwards, filtration with the moving window combined with logical conditions and the neighbourhood test was used. The cells for which anomalies were detected were reclassified into opposite classes. The distribution parameters were fitted based on the heights in the cells marked as water cells during the segmentation process. The areas for which most cells in the segmented DEM were classified as water areas were found using the movable window method. The threshold value was calculated based on the mean value and the standard deviation of the water pixel height inside the window. Next, each land pixel was checked for anomalies. If the land cell’s height was lower than the threshold value, the class of the cell was converted into the water class. If it was not part of the distribution (its value was higher than *T*), there was a chance that it was a land pixel that belongs to the land area contained in the neighbouring window or that it was an outlier recorded in the water area. For this reason, the neighbourhood test was used. Four windows were created and the percentage of land cover in them was calculated. If two or three neighbouring windows contained at least 90% of cells classified as land cells, the cell won the majority of votes and remained in the land class. On the other hand, if one or four windows contained at least 90% of the land class cells, then the authors concluded that there was a high probability that the tested cell was an outlier, and its class was changed to water. The authors of this review of the literature are of the opinion that the criterion used is not fully clear, as it appears incomprehensible to change the class in a situation where there are four windows satisfying the above condition. Unfortunately, the discussed paper seems to fail to explain this procedure and only focuses on demonstrating its efficiency.

Then, the opening and closing morphological operations were carried out in order to remove the remaining artefacts, such as gaps between the neighbouring land areas or disrupted branching of water areas. The operations were performed for the DEM parts encompassed by the window. The authors recommend that the size of the window should match the size of gaps between the areas. The distribution of pixels in both classes was calculated for the DEM part encompassed by the window, which was followed by the performance of the appropriate morphological transformation. This stage of the algorithm is supervised in nature (i.e., the areas, the morphological transformation, and the window size must be selected manually based on a visual analysis and expert knowledge of the authors).

The next step involved the use of the Hough transform [43] to remove the structures of anthropogenic nature. The Hough transform allows objects of a particular shape, in this case, a line, to be detected in the raster. As pointed out in the discussed paper, such application of Hough transform carries the possibility of removing hardly differentiated (linear) sections of the shoreline. For this reason, the threshold value for the number of peaks detected in the Hough space was lowered. Moreover, the minimum size of the detected line was also reduced as the anthropogenic structures may not form a perfect line in DEM and can be fragmented. Nevertheless, it is expected that the application of the Hough transform will generate many objects, not all of which are necessarily anthropogenic. For this reason, it is necessary to apply a criterion to enable the rejection of false candidates (e.g., linear shoreline sections) from among the actual anthropogenic structures (e.g., a pier, a platform). The applied rejection criterion involved shifting the moving column (or row) above the identified structure. The centre of the column (or row) must move above the pixels of the structure. In an idealised case, where the structure is part of the shore and its pixels are the edge pixels, the difference in pixel values before and after the centre of the column (or row) will amount to zero. This means that difference results significantly different from zero can be used to remove the anthropogenic structures.

In order to further smooth the obtained boundary, a convolution operation with the Gaussian kernel was used. Subsequently, the edge between the land and water areas was extracted. The obtained shoreline was superimposed on an aerial photo in order to visually assess the extraction process results.

Due to the lack of control points, only a visual assessment of the results of shoreline extraction using two different methods was performed. For a complete assessment of the proposed algorithm, it would be necessary to acquire information on the actual shoreline position (e.g., by performing measurement using a pole with a GPS receiver [44]), and then compare the results obtained using it with the results obtained by other methods. It is important to conduct the comparison using a common dataset, or even multiple common datasets, in order to take in to account the heterogeneity of water regions and the shoreline geometry.

### 3.8. Morphological Method (Yousef et al., 2013b [31])

This article [31] addresses most of the concerns regarding its predecessor. Additionally, it also contains an improved validation scheme, during which quantitative error was used alongside visual assessment and proposed methods were compared against two reference methods. This paper contains a different version of the reclassification algorithm. However, the changes were not clearly addressed in the manuscript. Therefore, it is unclear if the method was modified or if the previously published version [30] contained a mistake. In contrast to the earlier version, the pixels are only reclassified where only a single connection exists with an area, where at least 90% of pixels of the same category are detected. Pixels are not reclassified when more than one connection is detected. The publication does not clarify what happens when the number of connections is equal to zero. Arguably, the equality sign for single connection should be replaced with the less than or equal to sign.

Algorithm validation was validated on a previously used dataset and one additional dataset. Quantitative assessment of the shoreline extraction process was performed. Both proposed methods were compared, namely, the morphological algorithm and the method based on the SVM, as well as the reference methods for which the methods described in studies by Liu et al. [29] and Lee et al. [32] were chosen. Both the SVM method and the method from Lee et al. [32] used information from additional sensors for the extraction. These were, respectively, aerial photos and orthophotomap. The extraction results obtained by the individual methods were compared with the shoreline obtained using manual extraction based on aerial photos. The visual comparison involved superimposing the obtained shorelines and the manually obtained shoreline onto a common raster. Great extraction errors can be observed when using the method of Liu et al. [29], which may be related to the issue of setting the parameters that were not tested on other datasets.

## 4. Discussion and Conclusions

Before proceeding to compare the errors of individual methods, it is worth noting that the methods were assessed on different datasets, the measurements were conducted using different devices, the water areas differed in geometrical and optical properties, the shorelines had different geometries, and the extent of anthropogenic transformation, as well as the fact that the methods proposed by the authors were compared with a wide range of reference methods, of which few overlapped between the studies. Thus, an unambiguous assessment of the methods is, in the authors’ opinion, much more difficult, if not impossible. However, it is possible to draw certain conclusions concerning the research in the field of shoreline extraction based on ALS.

Based on the review, it can be concluded that:

- Most shoreline extraction methods rely on the vertical tidal system data. This dependency constraints their application in areas with different mean water levels;
- The Xu et al. method [25] does not consider tidal system data. However, it requires optimisation of underlying parameters for different waterbodies, complicating its practical application;
- Various reference methods, accuracy metrics, and validation techniques are used across studies [27,45,46]. This variance stresses the need for curated, accessible data repositories and standardised reference and validation protocols;
- Three out of seven investigated papers rely only on visual comparison for validation, further underscoring the need for open-access data repositories;
- Comparative studies on LiDAR-based shoreline extraction methods are scarce [27], indicating a research gap;
- Sensor differences in data collection can influence the results, a factor that should be considered in comparative studies;
- The use of standard reference methods is required for method comparison. Potential standards could include the methods described by Stockdonf et al. [20], Farris et al. [27], or Liu et al. [29];
- Broadened distribution of shoreline extraction methods is needed. This can be achieved through method performance descriptions in particular software [27], code sharing, or distribution of method implementation [29,32];
- Liu et al. [29] were the only authors to share their method as an ArcGIS extension. Farris et al. [27] also addressed this issue and planned to share their code. This observation highlights the need for popularising open science to facilitate meta-analyses and method verification [47,48,49];
- The absence of shared source code in the discussed studies supports the importance of open science initiatives such as National Aeronautics and Space Administration (NASA) Transform to OPen Science (TOPS) [50] and can also lead to the availability of ready-made implementations of particular methods [27,29];
- Outdated links to used datasets in many studies [27,29] underline the need for careful repository reorganisation to maintain the validity of older references [45,46];
- All discussed methods require human supervision or prior parameterisation [25,29], calling for research in automated shoreline extraction;
- During the review, no approaches utilising LiDAR data and modern machine learning techniques for shoreline extraction were found, indicating a gap in the field;
- Under specific circumstances, such as extremely high or low water levels, high wave action, unusual optical characteristics of the waterbody, and marshy shoreline, low height above sea level can significantly compromise the extraction process. This highlights the importance of further research that specifically targets these extreme cases.

## Figures and Tables

**Table 1:** The list of publications discussed in this review and summary of methods used for their validation.

Authors and Year	Method Validation
Farris et al., 2018 [27]	Discussion and comparison of three shoreline extraction methods: contour, grid, and profile. Visual comparison of differences between the methods. Quantitative comparison of differences by interpolating shoreline coordinates to transverse profiles spaced along the shoreline every 50 m. Calculation of mean differences in shoreline positions, root-mean-square (RMS) differences in shoreline positions and RMS differences in shoreline positions after the mean difference for each combination of methods. Statistical analysis of the uncertainty of results for the grid method and profiles.
Hua et al., 2021 [28]	Visual assessment of results on one dataset. Authors used the contour method as their reference method. However, they did not specify the source of reference method (possibly [20], described also in [27]).
Liu et al., 2011 [29]	The article presents two novel methods for shoreline extraction. The methods are validated against one common dataset. Performance assessment is based on visual analysis of the obtained shoreline.
Luque et al., 2012 [26]	In the paper, visual and quantitative assessment of the proposed method is carried out. One dataset is used for method validation. Obtained results are compared with results achieved with the contour method [20,27]. Additionally, uncertainty analysis of shoreline extraction with both methods is performed.
Xu et al., 2019 [25]	Visual and quantitative evaluation of shoreline extraction. Comparison of extraction results on five datasets. Calculation of accuracy metrics (correctness and completeness). The comparison was made against manually determined shorelines. The results were related to four other works, but only the level of accuracy (obtained on different datasets) was compared.
Yousef et al., 2013a [30]	The publication proposes visual assessment of shoreline extraction using the morphological algorithm. Two datasets were used to assess the method performance. Achieved results were not referenced against any other shoreline extraction methods.
Yousef et al., 2013b [31]	This work can be treated as a follow-up to the above article. Two datasets from the previous study and one new were used for validation. Qualitative assessment of the shoreline extraction method was carried out. To this end, cross-shore profiles were calculated alongside a manually derived shoreline. Obtained results were compared with the results obtained using Liu et al. method [29], Lee et al. method [32], and their own method based on the support vector regression (SVR) [31]. The two latter methods use data obtained with different sensors (i.e., aerial photos, orthoimagery). In addition to the above, the authors carried out estimation of errors and standard deviations for both of their methods using the Monte Carlo simulation.

**Table 2:** Methods, assumptions, and techniques used.

Method	Main Assumptions	Main Stages
Contour [27]	During data recording, the water level cannot exceed the mean high water (MHW) level; According to Farris et al. [27], in order to obtain satisfactory extraction results, a relatively simple morphology of the beach is required.	Creation of a contour for a specific reference height.
Grid [27]	During data recording, the water level cannot exceed the MHW level.	Rotation by the shoreline angle; Interpolation to a raster; Hanning filtering; A probabilistic approach to the estimation of the shoreline position.
Modified profile [27]	It only uses LiDAR data within the window surrounding the transverse profile; The distance between profiles defines the scale of the shoreline components taken into account during the extraction [20]; Results presented by Luque et al. [26] may indicate that a linear course of the share is required to obtain satisfactory extraction results.	Calculation of transverse profiles; Filtration with the threshold height value in relation to the zero height; Linear regression of individual point’s heights.
Anthropogenic [28]	The method only enables the detection of shores of anthropogenic nature; It requires the determination of both the shoreline position and the scan’s direction; Areas, including reflections from water, are characterised by a lower spatial density of reflections; Areas containing reflections from water are characterised by a lower mean height; A single scan of the area (a single flight) is assumed.	The use of information on the shoreline orientation; The use of information in the direction of flight during the scan; The use of density and height characteristics to identify water reflections; The authors’ original method for densifying the obtained point cloud.
Contour [29]	The reflection from the water surface is located at the reference height, e.g., MHW level.	The use of contouring operations for reflections found at the MHW level; Filtration of individual sections based on the section’s length threshold value; Extraction of the resulting curve; Simplification of the obtained line by the Douglas–Peucker method or the method for identifying the strongest curves in the shoreline in order to remove or extend some of them.
Object-oriented [29]	Reflections derived from the water surface are located at a height equal to or lower than the reference height, e.g., MHW level.	Median filter; DEM segmentation to land and water classes based on the local tidal system; Morphological operations; Filtration of particular clusters (objects) using the cluster size threshold value; Extraction of edges from a binary raster; Simplification of the obtained line by the Douglas–Peucker method or the method for identifying the strongest curves in the shoreline in order to remove or extend some of them.
EGTP [26]	Requires the specification of the zero (reference) height for a particular reference system.	Iterative extrapolation based on the height gradient.
Cost optimisation [25]	Points belonging to water areas form relatively flat areas in the point cloud; Moreover, they are areas with a low point density and a low height above the nearest fitted plane, which allows them to be distinguished from the flat components of land areas; The shoreline should not be located far from detected waterbodies; When choosing between multiple candidates, the shoreline with the least complexity is the appropriate candidate.	Plane fitting by the random sample consensus (RANSAC) method; Verification based on the density and height characteristics from the study by Smeeckaert et al. [23]; Cost optimisation model; The threshold distance between the point and the waterbody.
Morphological [30,31]	The local tidal system is available; Land areas should be located above the level determined by the local tidal system; It is assumed that the height distribution for land and water points follow the Gaussian distribution in both cases; Onshore anthropogenic structures are often linear objects, but unlike the linear sections of the shore, their neighbourhood contains water pixels.	DEM segmentation to land and water classes, based on the local tidal system; Morphological opening and closing operations; The Hough transform for the removal of linear anthropogenic structures; Detection of anomalies based on the assumption of the normal height distribution and the neighbourhood test.

**Table 3:** Data, methods, and their availability.

Authors and Year	Data Availability	Method Availability
Farris et al., 2018 [27]	Source of data (National Oceanic and Atmospheric Administration (NOAA) and United States Geological Survey (USGS) repositories) was provided. However, only the USGS repository is still available under this URL.	Code was not shared in any way, but the authors state plans to do so in the future. Information on used software (ArcGIS and MATLAB) was provided. A detailed description of execution of one of the methods in the ArcGIS software was provided.
Hua et al., 2021 [28]	Source of data was provided without providing means to access the data.	Code was not shared in any way. Only brief information on language used (C++) and the libraries used (PCL) was provided. Software “used for visualisation of point cloud” was mentioned, without providing its exact name.
Liu et al., 2011 [29]	Source of data and a detailed description (e.g., LiDAR used, measurement location) was provided. Data were not publicly shared.	Code was not shared, but the proposed method was made available as an ArcGIS plugin named “ShorelineExtractor”. Information on used software (ArcGIS, C++, Visual Basic. NET) was provided. Detailed description of method execution using GUI of the plugin was provided.
Luque et al., 2012 [26]	Source of data and a detailed description (e.g., LiDAR used, measurement location) was provided. Data were not publicly shared.	Code was not shared in any way. Information on used hardware and software (digital shoreline analysis system (DSAS), TerraMatch, TerraScan) was provided.
Xu et al., 2019 [25]	Valid URLs to open-source datasets used in the work were provided.	Code was not shared in any way. Information on used hardware and software (MATLAB) was provided.
Yousef et al., 2013a [30]	Source of data (NOAA repositories) was provided, but the repository is no longer available under that URL.	Code was not shared in any way. No information on the program used was provided, with the exception of using the VDatum software for coordinate transformation.
Yousef et al., 2013b [31]	Source of data (NOAA repositories) was provided, but the repository is no longer available under that URL.	Code was not shared in any way. Information on used hardware and software (ArcGIS, MATLAB, VDatum) was provided.

**Table 4:** Three main methods used by Farris et al., 2018 [27].

Method	Description
Contour	Allows a contour of the MHW level to be obtained by using the contour obtaining operation in the ArcGIS software.
Grid	A method based on the interpolation of heights onto a square grid.
Profile	A modified profile method described by Stockdonf et al. [20]. It uses a 20 m wide window determined along transverse profiles.

**Table 5:** Differences in RMS error of shoreline positions estimation using different methods as reported by Farris et al., 2018 [27].

Method	Grid	Profile20	Profile10	Contour	Smoothed Contour Bend Simplify
Grid	-	-	-	-	-
Profile20	0.54 m	-	-	-	-
Profile10	0.55 m	0.48 m	-	-	-
Contour	0.64 m	0.87 m	0.88 m	-	-
Smoothed contour bend simplify	0.67 m	0.91 m	0.89 m	0.62 m	-
Smoothed contour smooth line	0.47 m	0.75 m	0.77 m	0.34 m	0.48 m

**Table 6:** Advantages and disadvantages of compared methods as reported by Farris et al., 2018 [27].

Method	Advantages	Disadvantages
Contour	Easy to transfer to other users due to the possibility of performing it using the ArcGIS software; A similar method can be performed using other similar GIS-type programs; High efficiency of shoreline extraction.	Limited ability to perform the extraction of shorelines with complex geometry; Not suitable for the application to datasets for which the water level is high, partially exceeding the MHW level; Ensures only a global estimation of uncertainty.
Grid	Faster than the profile method; Uses a higher percentage of data than the profile method; The possibility of using the method in the smoothing version allows a smoother shoreline to be obtained; Provides information on the uncertainty of individual points.	Limited ability to perform the extraction of shorelines with complex geometry; Not suitable for the application to datasets for which the water level is high, partially exceeding the MHW level; Requires both expert knowledge of a programming language and the performance of independent implementation.
Profile	Provides a shoreline with the possibility of performing quality control, which enables the incorporation of results obtained by this method into long-term databases; Enables the extrapolation of data collected during high water, while additionally allowing the uncertainty related to the extrapolation to be calculated; Enables the performance of the extraction of shorelines with complex geometry; Provides information on the uncertainty of individual points.	More time consuming than the grid method; Only uses the reflections derived from the window defined by users and covers the area around profiles; Requires both expert knowledge of a programming language and the performance of independent implementation.

**Table 7:** Comparison of achieved accuracy level and number of used datasets as reported by Xu et al., 2019 [25].

Authors and Year	Input Data Type	Accuracy Level (m)	Number of Datasets Used during Validation
Niedermeier et al., 2000 [38]	Image	31	1
Di et al., 2003 [39]	Image	8.5	4
Liu et al., 2007 [40]	Point cloud	4.5	1
Lee et al., 2010 [41]	Point cloud	1.5	4
Xu et al., 2019 [25]	Point cloud	1	5

**Table 8:** Parameters of the cost optimisation model proposed by Xu et al., 2019 [25].

Stage	Parameter	Range	Suggested Value	Unit
Removal of waterbodies	*n_p_*	500–10,000	500	pt
	*T_e_*	0.5–5	2	m
	*T_D_*	0.1–0.5	0.3	pt/m^2^
Shoreline optimisation	*k*	20–100	50	pt
	*T_d_*	0.5–3	2	m
	λ	1–10	3	–
	*T_b_*	0.1–1	0.5	m
